# Supplementary material for: A distributional reinforcement learning model for optimal glucose control after cardiac surgery
Source: NPJ Digit Med. 2025 May 27;8:313. doi: 10.1038/s41746-025-01709-9 (PMC12116759; doi:10.1038/s41746-025-01709-9)
Supplement: Supplementary file 1 — Supplement [file 41746_2025_1709_MOESM1_ESM.pdf]

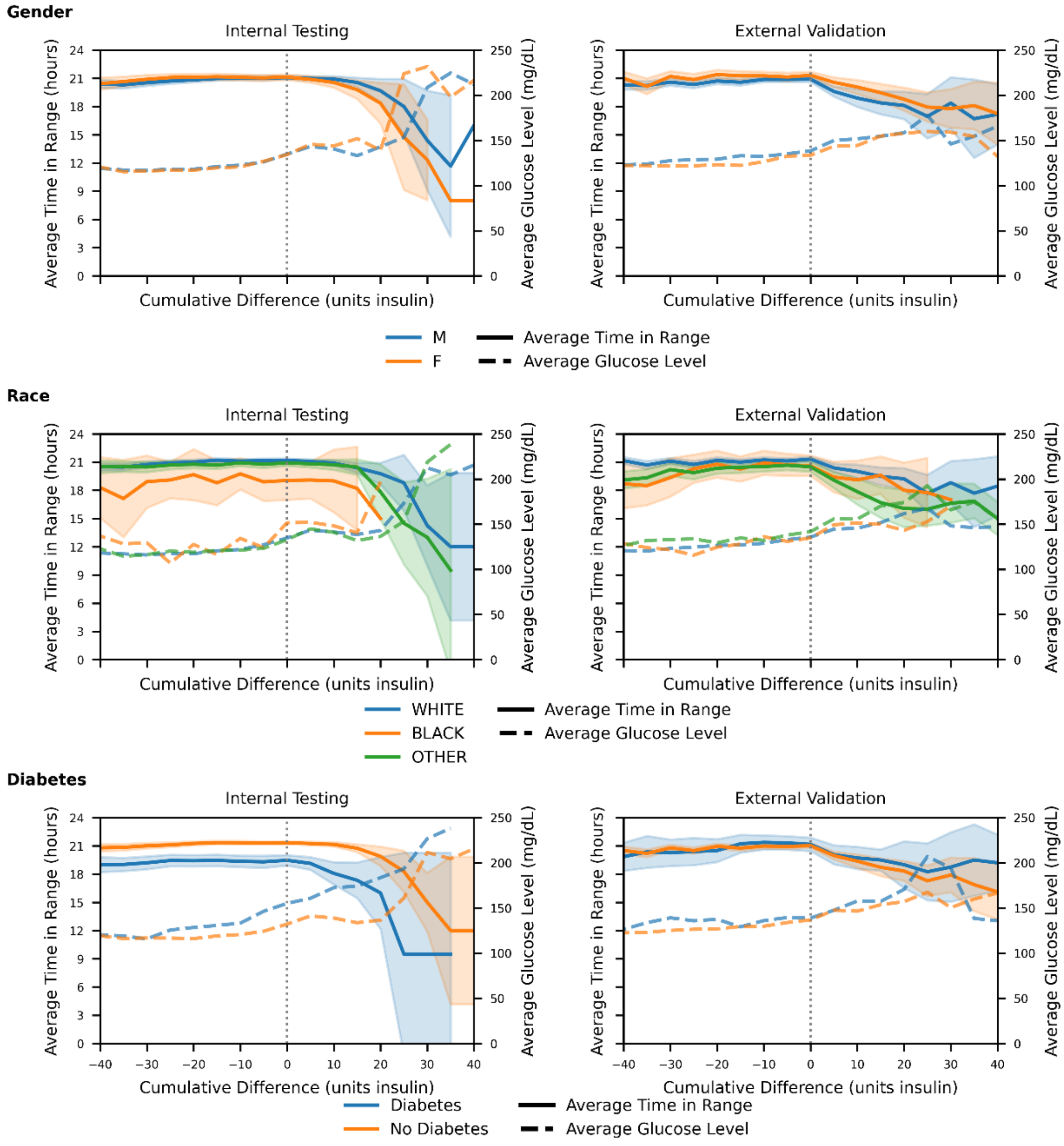

Supplementary Figure 1. Time in range analysis stratified by subgroup. Average time in range ± the standard error of the mean shown via solid lines. Dashed lines indicate the average glucose level corresponding to that subgroup.

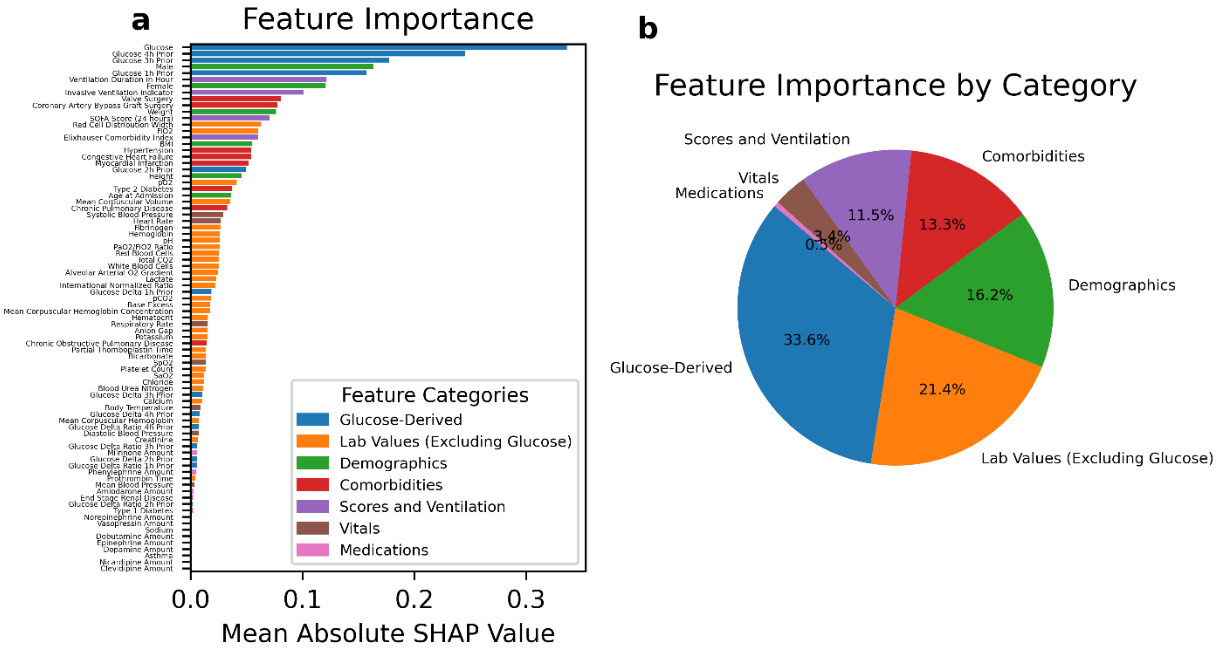

Supplementary Figure 2. Feature importances for GLUOCSE. **a)** Ranked feature importances by mean absolute SHAP value. **b)** Relative contributions of varying categorizations of features.

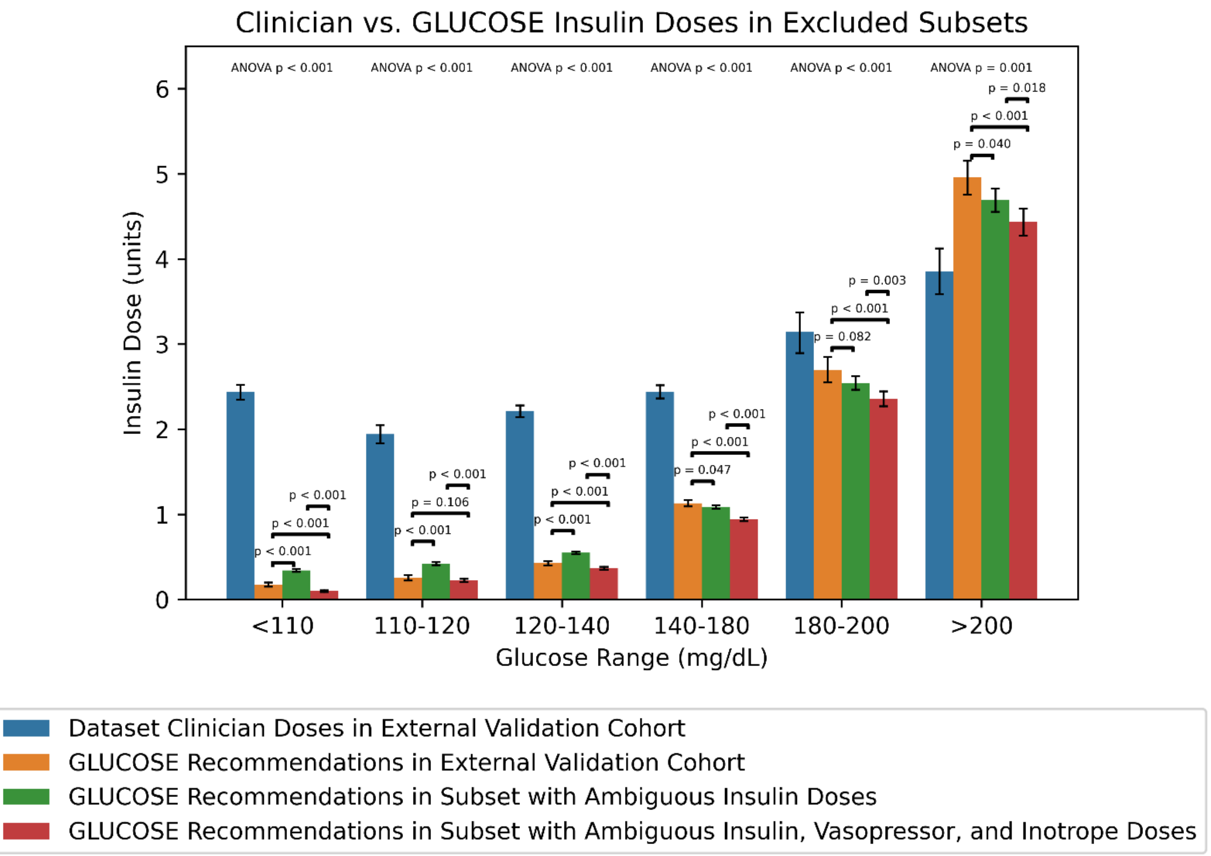

Supplementary Figure 3. Analysis of GLUCOSE predictions in excluded subsets. Average insulin values in varying clinically relevant ranges are shown in each of the subsets. 95% confidence intervals are shown for each bar.

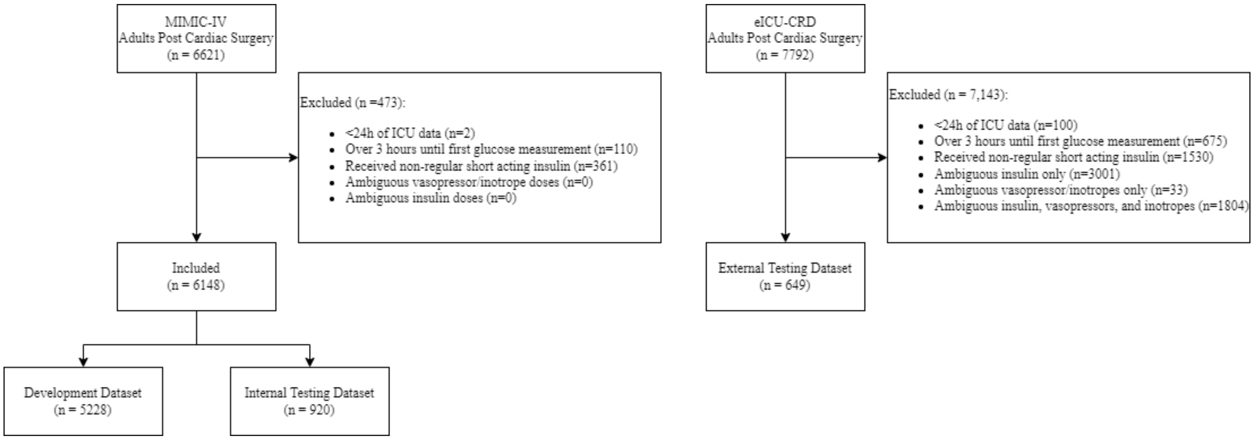

Supplementary Figure 4. CONSORT diagram. Inclusion/exclusion criteria applied during data preprocessing.

Supplementary Table 1. Baseline population characteristics. Characteristics of all patients used in the primary analysis across demographic information, vitals, laboratory data, scores, and medications.

		Development Cohort	External Validation Cohort	P-Value
**Admission Age, median [Q1, Q3]**		68.0 [61.0,76.0]	67.0 [60.0,75.0]	0.093
**Gender, n (%)**	**Female**	1746 (28.4)	213 (32.8)	0.02
	**Male**	4402 (71.6)	436 (67.2)	
**Race, n (%)**	**Asian**	136 (2.2)	15 (2.3)	<0.001
	**Black**	222 (3.6)	51 (7.9)	
	**Hispanic**	175 (2.8)	79 (12.2)	
	**Native American**	11 (0.2)		
	**Other**	219 (3.6)		
	**Unknown**	841 (13.7)	72 (11.1)	
	**White**	4544 (73.9)	432 (66.6)	
**Height, median [Q1,Q3]**		173.0 [165.0,178.0]	170.2 [162.6,177.8]	0.059
**Weight, median [Q1,Q3]**		83.2 [72.0,96.0]	85.6 [72.6,99.7]	0.005
**BMI, median [Q1,Q3]**		28.2 [25.1,32.1]	29.2 [25.8,33.9]	<0.001
**Type 1 Diabetes, n (%)**		33 (0.5)	2 (0.3)	0.77
**Type 2 Diabetes, n (%)**		868 (14.1)	59 (9.1)	<0.001
**Hypertension, n (%)**		3814 (62.0)	177 (27.3)	<0.001
**End Stage Renal Disease, n (%)**		131 (2.1)	17 (2.6)	0.503
**Chronic Obstructive Pulmonary Disease, n (%)**		297 (4.8)	65 (10.0)	<0.001
**Chronic Pulmonary Disease, n (%)**		1036 (16.9)	40 (6.2)	<0.001
**Asthma, n (%)**		1 (0.0)	29 (4.5)	<0.001
**History of Myocardial Infarct, n (%)**		1782 (29.0)	56 (8.6)	<0.001
**Congestive Heart Failure, n (%)**		1665 (27.1)	52 (8.0)	<0.001
**SaO2, median [Q1,Q3]**		97.0 [96.0,98.0]	98.0 [96.0,99.0]	<0.001
**pO2, median [Q1,Q3]**		174.0 [113.0,300.0]	111.0 [86.0,153.0]	<0.001
**pCO2, median [Q1,Q3]**		40.0 [36.0,44.0]	40.4 [36.4,44.8]	0.527
**FIO2, median [Q1,Q3]**		50.0 [50.0,80.0]	50.0 [40.0,60.0]	<0.001
**AaDO2, median [Q1,Q3]**		200.8 [133.5,323.0]	168.8 [125.2,269.2]	<0.001
**paO2 FIO2 ratio, median [Q1,Q3]**		244.1 [180.0,322.5]	230.2 [171.0,304.1]	<0.001
**pH, median [Q1,Q3]**		7.4 [7.3,7.4]	7.4 [7.4,7.4]	<0.001
**Base Excess, median [Q1,Q3]**		-1.0 [-3.0,0.0]	-2.0 [-4.0,0.0]	<0.001
**Bicarbonate, median [Q1,Q3]**		23.0 [22.0,25.0]	24.0 [22.0,25.0]	<0.001
**Total CO2, median [Q1,Q3]**		25.0 [23.0,26.0]	24.2 [22.0,26.0]	0.265
**Hematocrit, median [Q1,Q3]**		29.8 [26.8,32.9]	29.3 [26.0,33.0]	0.013
**Hemoglobin, median [Q1,Q3]**		10.0 [8.9,11.1]	9.9 [8.8,11.1]	0.094
**Chloride, median [Q1,Q3]**		107.0 [105.0,110.0]	109.0 [107.0,112.0]	<0.001
**Calcium, median [Q1,Q3]**		8.2 [7.9,8.5]	8.1 [7.7,8.5]	<0.001
**Temperature, median [Q1,Q3]**		36.8 [36.3,37.2]	37.1 [36.7,37.5]	<0.001
**Potassium, median [Q1,Q3]**		4.4 [4.1,4.7]	4.2 [3.9,4.5]	<0.001

**Sodium, median [Q1,Q3]**		138.0 [137.0,140.0]	141.0 [138.0,143.0]	<0.001
**Lactate, median [Q1,Q3]**		2.0 [1.6,2.7]	2.1 [1.7,2.6]	<0.001
**Glucose, median [Q1,Q3]**		124.0 [108.0,145.0]	130.0 [113.0,150.0]	<0.001
**WBC, median [Q1,Q3]**		13.1 [10.4,16.9]	13.1 [10.7,16.7]	0.071
**MCH, median [Q1,Q3]**		30.5 [29.3,31.6]	30.0 [28.9,31.1]	<0.001
**MCHC, median [Q1,Q3]**		33.7 [32.8,34.6]	33.6 [32.9,34.1]	<0.001
**MCV, median [Q1,Q3]**		90.0 [87.0,93.0]	89.4 [86.1,92.7]	<0.001
**Platelets, median [Q1,Q3]**		144.0 [116.0,179.0]	144.0 [113.0,181.0]	0.668
**RBC, median [Q1,Q3]**		3.3 [2.9,3.7]	3.3 [3.0,3.8]	<0.001
**RDW, median [Q1,Q3]**		13.7 [13.0,14.7]	14.2 [13.5,15.4]	<0.001
**Anion Gap, median [Q1,Q3]**		11.0 [10.0,13.0]	8.0 [6.0,10.0]	<0.001
**BUN, median [Q1,Q3]**		16.0 [13.0,20.0]	17.0 [13.0,22.0]	<0.001
**Creatinine, median [Q1,Q3]**		0.9 [0.7,1.1]	1.0 [0.8,1.2]	<0.001
**Fibrinogen, median [Q1,Q3]**		201.0 [167.0,246.0]	210.0 [173.0,270.0]	0.109
**INR, median [Q1,Q3]**		1.3 [1.2,1.4]	1.2 [1.1,1.4]	<0.001
**PT, median [Q1,Q3]**		14.2 [13.1,15.7]	13.4 [12.1,16.1]	<0.001
**PTT, median [Q1,Q3]**		30.3 [27.4,34.9]	31.7 [28.4,35.3]	0.001
**Heart Rate, median [Q1,Q3]**		81.0 [75.0,89.0]	86.0 [77.0,93.0]	<0.001
**SBP, median [Q1,Q3]**		111.0 [101.0,121.0]	115.0 [104.0,128.0]	<0.001
**DBP, median [Q1,Q3]**		56.0 [50.0,62.7]	55.0 [49.0,62.0]	<0.001
**MBP, median [Q1,Q3]**		73.0 [66.0,80.0]	74.0 [67.0,81.0]	<0.001
**Respiratory Rate, median [Q1,Q3]**		17.0 [14.0,20.0]	18.0 [14.0,22.0]	<0.001
**SpO2, median [Q1,Q3]**		99.0 [96.0,100.0]	98.0 [96.0,100.0]	<0.001
**24h SOFA Score, median [Q1,Q3]**		5.0 [3.0,7.0]	6.0 [4.0,8.0]	<0.001
**Elixhauser, median [Q1,Q3]**		4.0 [3.0,6.0]	0.0 [0.0,2.0]	<0.001
**Invasive Ventilation in Hour**	**0**	79626 (57.2)	1039 (7.0)	<0.001
	**1**	59623 (42.8)	13769 (93.0)	
**Minutes on Ventilator in Hour, median [Q1,Q3]**		0.0 [0.0,60.0]	60.0 [60.0,60.0]	<0.001
**Dopamine Amount, median [Q1,Q3]**		0.0 [0.0,0.0]	0.0 [0.0,0.0]	<0.001
**Epinephrine Amount, median [Q1,Q3]**		0.0 [0.0,0.0]	0.0 [0.0,0.0]	0.052
**Norepinephrine Amount, median [Q1,Q3]**		0.0 [0.0,0.0]	0.4 [0.0,3.5]	<0.001
**Phenylephrine Amount, median [Q1,Q3]**		3.7 [0.0,22.2]	0.0 [0.0,0.0]	<0.001
**Vasopressin Amount, median [Q1,Q3]**		0.0 [0.0,0.0]	0.0 [0.0,0.0]	0.199
**Amiodarone Amount, median [Q1,Q3]**		0.0 [0.0,0.0]	0.0 [0.0,0.0]	<0.001
**Clevidipine Amount, median [Q1,Q3]**		0.0 [0.0,0.0]	0.0 [0.0,0.0]	0.329
**Dobutamine Amount, median [Q1,Q3]**		0.0 [0.0,0.0]	0.0 [0.0,0.0]	<0.001
**Milrinone Amount, median [Q1,Q3]**		0.0 [0.0,0.0]	0.0 [0.0,0.0]	0.001
**Nicardipine Amount, median [Q1,Q3]**		0.0 [0.0,0.0]	0.0 [0.0,0.0]	<0.001

Supplementary Table 2. Population characteristics of excluded external validation patients. Characteristics of all patients excluded from external validation due to ambiguous medication information. Information across demographic information, vitals, laboratory data, scores, and medications shown.

		Subset with Ambiguous Insulin Doses	Subset with Ambiguous Insulin, Vasopressor, and Inotrope Doses	Primary External Validation Cohort	P-Value
**n**		3001	1804	649	
**Admission Age, mean (SD)**		67.0 (12.4)	67.6 (11.5)	67.0 (11.1)	0.27
**Gender, n (%)**	**Female**	992 (33.1)	584 (32.4)	213 (32.8)	0.888
	**Male**	2009 (66.9)	1220 (67.6)	436 (67.2)	
**Race, n (%)**	**Asian**	97 (3.2)	9 (0.5)	15 (2.3)	<0.001
	**Black**	235 (7.8)	43 (2.4)	51 (7.9)	
	**Hispanic**	79 (2.6)	50 (2.8)	79 (12.2)	
	**Unkown**	156 (5.2)	84 (4.7)	72 (11.1)	
	**White**	2434 (81.1)	1618 (89.7)	432 (66.6)	
**Height, mean (SD)**		171.2 (11.6)	171.7 (10.9)	170.3 (10.2)	0.029
**Weight, mean (SD)**		86.4 (22.4)	87.0 (20.4)	87.8 (21.4)	0.262
**BMI, mean (SD)**		29.2 (6.4)	29.5 (6.4)	30.1 (6.3)	0.005
**Type 1 Diabetes, n (%)**		8 (0.3)	5 (0.3)	2 (0.3)	0.983
**Type 2 Diabetes, n (%)**		93 (3.1)	22 (1.2)	59 (9.1)	<0.001
**Hypertension, n (%)**		750 (25.0)	121 (6.7)	177 (27.3)	<0.001
**End Stage Renal Disease, n (%)**		58 (1.9)	25 (1.4)	17 (2.6)	0.111
**Chronic Obstructive Pulmonary Disease, n (%)**		290 (9.7)	176 (9.8)	65 (10.0)	0.962
**Chronic Pulmonary Disease, n (%)**		212 (7.1)	45 (2.5)	40 (6.2)	<0.001
**Asthma, n (%)**		146 (4.9)	108 (6.0)	29 (4.5)	0.161
**History of Myocardial Infarct, n (%)**		148 (4.9)	98 (5.4)	56 (8.6)	0.001
**Congestive Heart Failure, n (%)**		236 (7.9)	97 (5.4)	52 (8.0)	0.003
**Glucose, mean (SD)**		132.2 (30.7)	130.6 (30.5)	134.5 (33.0)	<0.001
**Hyperglycemia Step Count, n (%)**	**Non-hyperglycemic**	64715 (94.6)	38877 (94.2)	13597 (91.8)	<0.001
	**Hyperglycemic**	3699 (5.4)	2401 (5.8)	1211 (8.2)	
**Hypoglycemia Step Count, n (%)**	**Non-hypoglycemic**	68221 (99.7)	41123 (99.6)	14739 (99.5)	<0.001
	**Hypoglycemic**	193 (0.3)	155 (0.4)	69 (0.5)	
**Glucose During Hyperglycemia, mean (SD)**		210.5 (47.4)	206.7 (28.4)	209.7 (28.8)	0.001
**Glucose During Hypoglycemia, mean (SD)**		60.0 (12.2)	64.0 (5.2)	60.9 (11.1)	0.001
**SaO2, median [Q1,Q3]**		97.0 [96.0,99.0]	97.0 [95.0,99.0]	98.0 [96.0,99.0]	<0.001
**pO2, median [Q1,Q3]**		109.0 [85.0,149.0]	105.0 [81.0,145.0]	111.0 [86.0,153.0]	<0.001
**pCO2, median [Q1,Q3]**		41.0 [37.0,45.0]	40.0 [36.0,44.0]	40.4 [36.4,44.8]	<0.001
**FIO2, median [Q1,Q3]**		40.0 [36.0,52.0]	45.0 [40.0,70.0]	50.0 [40.0,60.0]	<0.001

**AaDO2, median [Q1,Q3]**		143.0 [83.5,220.0]	161.7 [112.2,252.7]	168.8 [125.2,269.2]	<0.001
**paO2 FIO2 ratio, median [Q1,Q3]**		262.0 [190.0,367.5]	234.0 [172.5,316.1]	230.2 [171.0,304.1]	<0.001
**pH, median [Q1,Q3]**		7.4 [7.4,7.4]	7.4 [7.4,7.4]	7.4 [7.4,7.4]	<0.001
**Base Excess, median [Q1,Q3]**		-1.1 [-3.7,1.0]	-2.1 [-4.0,0.0]	-2.0 [-4.0,0.0]	<0.001
**Bicarbonate, median [Q1,Q3]**		24.0 [22.0,25.0]	23.0 [21.0,25.0]	24.0 [22.0,25.0]	<0.001
**Total CO2, median [Q1,Q3]**		24.0 [22.0,26.0]	24.0 [22.0,26.0]	24.2 [22.0,26.0]	<0.001
**Hematocrit, median [Q1,Q3]**		31.0 [27.3,34.6]	29.7 [26.5,33.2]	29.3 [26.0,33.0]	<0.001
**Hemoglobin, median [Q1,Q3]**		10.3 [9.1,11.6]	9.9 [8.8,11.1]	9.9 [8.8,11.1]	<0.001
**Chloride, median [Q1,Q3]**		108.0 [106.0,111.0]	109.0 [107.0,112.0]	109.0 [107.0,112.0]	<0.001
**Calcium, median [Q1,Q3]**		8.2 [7.8,8.6]	8.1 [7.7,8.6]	8.1 [7.7,8.5]	0.002
**Temperature, median [Q1,Q3]**		37.2 [36.7,37.6]	37.2 [36.7,37.6]	37.1 [36.7,37.5]	<0.001
**Potassium, median [Q1,Q3]**		4.2 [3.9,4.5]	4.2 [3.9,4.5]	4.2 [3.9,4.5]	<0.001
**Sodium, median [Q1,Q3]**		139.0 [137.0,142.0]	141.0 [139.0,143.0]	141.0 [138.0,143.0]	<0.001
**Lactate, median [Q1,Q3]**		2.0 [1.6,2.4]	2.0 [1.7,2.4]	2.1 [1.7,2.6]	<0.001
**Glucose, median [Q1,Q3]**		129.0 [114.0,146.0]	127.0 [110.0,147.0]	130.0 [113.0,150.0]	<0.001
**WBC, median [Q1,Q3]**		12.5 [9.5,16.2]	13.1 [10.1,16.6]	13.1 [10.4,16.7]	<0.001
**MCH, median [Q1,Q3]**		30.1 [28.9,31.3]	30.1 [29.1,31.2]	30.0 [28.9,31.1]	0.425
**MCHC, median [Q1,Q3]**		33.5 [32.8,34.2]	33.6 [32.8,34.3]	33.6 [32.9,34.1]	0.385
**MCV, median [Q1,Q3]**		89.7 [86.2,93.0]	89.6 [86.6,92.9]	89.4 [86.1,92.7]	0.17
**Platelets, median [Q1,Q3]**		138.0 [110.0,172.0]	135.0 [107.0,169.0]	144.0 [113.0,181.0]	<0.001
**RBC, median [Q1,Q3]**		3.5 [3.1,3.9]	3.4 [3.0,3.8]	3.3 [3.0,3.8]	<0.001
**RDW, median [Q1,Q3]**		14.2 [13.4,15.2]	14.3 [13.5,15.3]	14.2 [13.5,15.4]	<0.001
**Anion Gap, median [Q1,Q3]**		8.0 [6.0,11.5]	10.3 [8.0,13.0]	8.0 [6.0,10.0]	<0.001
**BUN, median [Q1,Q3]**		16.0 [13.0,22.0]	17.0 [13.0,22.0]	17.0 [13.0,22.0]	0.002
**Creatinine, median [Q1,Q3]**		1.0 [0.8,1.3]	1.0 [0.8,1.2]	1.0 [0.8,1.2]	0.294
**Fibrinogen, median [Q1,Q3]**		241.0 [193.0,306.2]	241.0 [197.8,299.2]	210.0 [173.0,270.0]	<0.001
**INR, median [Q1,Q3]**		1.2 [1.1,1.3]	1.4 [1.2,1.5]	1.2 [1.1,1.4]	<0.001
**PT, median [Q1,Q3]**		13.5 [11.9,15.8]	16.5 [15.2,17.9]	13.4 [12.1,16.1]	<0.001
**PTT, median [Q1,Q3]**		30.5 [27.4,34.9]	34.9 [31.0,40.0]	31.7 [28.4,35.3]	<0.001
**Heart Rate, median [Q1,Q3]**		80.0 [73.0,90.0]	81.0 [73.0,91.0]	86.0 [77.0,93.0]	<0.001
**SBP, median [Q1,Q3]**		116.0 [104.0,130.0]	114.0 [103.0,127.0]	115.0 [104.0,128.0]	<0.001
**DBP, median [Q1,Q3]**		55.0 [48.0,62.0]	54.0 [48.0,61.0]	55.0 [49.0,62.0]	<0.001
**MBP, median [Q1,Q3]**		74.0 [67.0,82.0]	73.0 [66.0,80.0]	74.0 [67.0,81.0]	<0.001
**Respiratory Rate, median [Q1,Q3]**		17.0 [14.0,21.0]	17.0 [14.0,21.0]	18.0 [14.0,22.0]	<0.001
**SpO2, median [Q1,Q3]**		98.0 [96.0,100.0]	98.0 [96.0,100.0]	98.0 [96.0,100.0]	<0.001
**24h SOFA Score, median [Q1,Q3]**		3.0 [1.0,5.0]	4.0 [1.0,5.0]	6.0 [4.0,8.0]	<0.001
**Elixhauser, median [Q1,Q3]**		1.0 [0.0,2.0]	0.0 [0.0,1.0]	0.0 [0.0,2.0]	<0.001
**Invasive Ventilation in Hour, median [Q1,Q3]**	**0**	19056 (27.9)	4507 (10.9)	1039 (7.0)	<0.001
	**1**	49358 (72.1)	36771 (89.1)	13769 (93.0)	
**Minutes on Ventilator Within Hour, median [Q1,Q3]**		60.0 [0.0,60.0]	60.0 [60.0,60.0]	60.0 [60.0,60.0]	<0.001

**Dopamine Amount, median [Q1,Q3]**		0.0 [0.0,0.0]	0.0 [0.0,0.0]	0.0 [0.0,0.0]	<0.001
**Epinephrine Amount, median [Q1,Q3]**		0.0 [0.0,0.0]	0.0 [0.0,0.0]	0.0 [0.0,0.0]	<0.001
**Norepinephrine Amount, median [Q1,Q3]**		0.0 [0.0,0.0]	0.0 [0.0,0.0]	0.4 [0.0,3.5]	<0.001
**Phenylephrine Amount, median [Q1,Q3]**		0.0 [0.0,0.0]	0.0 [0.0,0.0]	0.0 [0.0,0.0]	<0.001
**Vasopressin Amount, median [Q1,Q3]**		0.0 [0.0,0.0]	0.0 [0.0,0.0]	0.0 [0.0,0.0]	<0.001
**Amiodarone Amount, median [Q1,Q3]**		0.0 [0.0,0.0]	0.0 [0.0,0.0]	0.0 [0.0,0.0]	<0.001
**Clevidipine Amount, median [Q1,Q3]**		0.0 [0.0,0.0]	0.0 [0.0,0.0]	0.0 [0.0,0.0]	<0.001
**Dobutamine Amount, median [Q1,Q3]**		0.0 [0.0,0.0]	0.0 [0.0,0.0]	0.0 [0.0,0.0]	<0.001
**Milrinone Amount, median [Q1,Q3]**		0.0 [0.0,0.0]	0.0 [0.0,0.0]	0.0 [0.0,0.0]	<0.001
**Nicardipine Amount, median [Q1,Q3]**		0.0 [0.0,0.0]	0.0 [0.0,0.0]	0.0 [0.0,0.0]	<0.001

Supplemental Table 3. ICD-9-PCS and ICD-10-PCS codes. Table of codes used to identify CABG, valve repair, and valve replacement procedures.

Category	Code	Translation
CABG	361X	Aortocoronary Bypass for Heart Revascularization
	2100X	Coronary Artery, One Artery
	2110X	Coronary Artery, Two Arteries
	2120X	Coronary Artery, Three Arteries
	2130X	Coronary Artery, Four or More Arteries
Valve Repair/Replacement	352X	Open and other replacement of unspecified heart valve
	3511	Open heart valvuloplasty of aortic valve without replacement
	3512	Open heart valvuloplasty of mitral valve without replacement
	3513	Open heart valvuloplasty of pulmonary valve without replacement
	3514	Open heart valvuloplasty of tricuspid valve without replacement
	02RF0X	Replacement, Aortic Valve
	02RG0X	Replacement, Tricuspid Valve
	02RH0X	Replacement, Pulmonary Valve
	02RJ0X	Replacement, Tricuspid Valve
	02QF0X	Repair, Aortic Valve
	02QG0X	Repair, Mitral Valve
	02QH0X	Repair, Pulmonary Valve
	02QJ0X	Repair, Tricuspid Valve

Supplementary Table 4. Admission diagnosis reasons. Admission diagnosis reasons used in eICU-CRD in the absence of available ICD-9-PCS and ICD-10-PCS to identify post-cardiac surgery patient.

**Category**	**String**
CABG	admission diagnosis|All Diagnosis|Operative|Diagnosis|Cardiovascular|CABG, minimally invasive; mid-CABG
	admission diagnosis|All Diagnosis|Operative|Diagnosis|Cardiovascular|CABG alone, coronary artery bypass grafting
	admission diagnosis|All Diagnosis|Operative|Diagnosis|Cardiovascular|CABG alone, redo
	admission diagnosis|All Diagnosis|Operative|Diagnosis|Cardiovascular|CABG redo with other operation
Valve	admission diagnosis|All Diagnosis|Operative|Diagnosis|Cardiovascular|Pulmonary valve surgery
	admission diagnosis|All Diagnosis|Operative|Diagnosis|Cardiovascular|Aortic valve replacement (isolated)
	admission diagnosis|All Diagnosis|Operative|Diagnosis|Cardiovascular|Mitral valve repair
	admission diagnosis|All Diagnosis|Operative|Diagnosis|Cardiovascular|Aortic and Mitral valve replacement
	admission diagnosis|All Diagnosis|Operative|Diagnosis|Cardiovascular|Mitral valve replacement
	admission diagnosis|All Diagnosis|Operative|Diagnosis|Cardiovascular|Tricuspid valve surgery
Both	admission diagnosis|All Diagnosis|Operative|Diagnosis|Cardiovascular|CABG redo with valve repair/replacement
	admission diagnosis|All Diagnosis|Operative|Diagnosis|Cardiovascular|CABG with pulmonic or tricuspid valve repair or replacement ONLY
	admission diagnosis|All Diagnosis|Operative|Diagnosis|Cardiovascular|CABG with double valve repair/replacement
	admission diagnosis|All Diagnosis|Operative|Diagnosis|Cardiovascular|CABG with mitral valve replacement
	admission diagnosis|All Diagnosis|Operative|Diagnosis|Cardiovascular|CABG with aortic valve replacement
	admission diagnosis|All Diagnosis|Operative|Diagnosis|Cardiovascular|CABG with mitral valve repair
